# Systematic determination of muscle groups and optimal stimulation intensity for simultaneous TMS mapping of multiple muscles in the upper limb

**DOI:** 10.14814/phy2.15527

**Published:** 2022-12-02

**Authors:** Akiko Yuasa, Shintaro Uehara, Yusuke Sawada, Yohei Otaka

**Affiliations:** ^1^ Department of Rehabilitation Medicine I Fujita Health University School of Medicine Toyoake Aichi Japan; ^2^ Faculty of Rehabilitation Fujita Health University School of Health Sciences Toyoake Aichi Japan; ^3^ Fujita Health University Nanakuri Memorial Hospital Tsu Mie Japan

**Keywords:** cortical representation, motor cortex, motor map, multiple muscles, transcranial magnetic stimulation, upper limb

## Abstract

Transcranial magnetic stimulation has been used to assess plastic changes in the cortical motor representations of targeted muscles. The present study explored the optimal settings and stimulation intensity for simultaneous motor mapping of multiple upper‐limb muscles across segments. In 15 healthy volunteers, we evaluated cortical representations simultaneously from one muscle in the shoulder, two in the upper arm, two in the forearm, and two intrinsic hand muscles, using five stimulation intensities, ranging from 40% to 100% of the maximum stimulator output. We represented the motor map area acquired at each intensity as a percentage of the maximum for each muscle. We defined a motor map area between 25% and 75% of the maximum as the optimal area size with sufficient scope for both up‐ and down‐regulation, and stimulation intensities producing the map area size within this range as the optimal intensities. We found that motor maps with optimal area sizes could be produced simultaneously for the four distal muscles of the forearm and hand in most participants when the stimulation intensity was set at 120–140% of the resting motor threshold (RMT) of the first dorsal interosseous. For the remaining three proximal muscles, motor maps with optimal area sizes were produced only in a few participants, even when using a higher intensity (180–220% RMT). These findings suggest that cortical representations can be assessed simultaneously in a group of distal muscles using a relatively low stimulation intensity, while a separate operation is required to assess that of the proximal muscles.

## Graphical abstract

We computed the range of stimulation intensity that produced motor maps with an optimal area size for each of the seven upper‐limb muscles. Results showed that optimal area sizes could be produced simultaneously for the four distal muscles of the forearm and hand using a relatively low stimulation intensity. For proximal muscles in the shoulder and upper arm, a markedly higher stimulation intensity was required, and the optimal area size was produced in only a few participants.

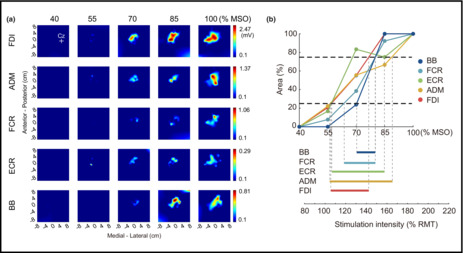

## 1 INTRODUCTION

In the last few decades, motor mapping with transcranial magnetic stimulation (TMS) has been used to noninvasively measure the cortical representation in the human primary motor cortex (Pascual‐Leone et al., 1993, 1995; Van De Ruit et al., 2015; Wassermann et al., 1992). Several parameters of motor maps [i.e., area, volume, and center of gravity (COG)] have been commonly used to assess the modulation of cortical motor outputs (i.e., reorganization of motor cortex) after certain events. Many studies have revealed the association between motor map area changes and motor skill learning or functional improvements. For example, previous TMS studies have shown that motor map area of a targeted muscle has increased after acquiring motor skills that involve that muscle in humans (Kleim et al., 2007; Pascual‐Leone et al., 1995; Tyč et al., 2005). The changes in motor map area have also been demonstrated in patients with neurological disorders, such as stroke. In the early phase of stroke, the size of the motor map area decreases in the affected hemisphere, and then increases along with functional improvements achieved with rehabilitative training (Boake et al., 2007; Platz et al., 2005; Yarossi et al., 2014). Moreover, another study showed that 16 weeks of immobilization of the ankle joint resulted in a decrease in the motor map area (Pascual‐Leone et al., 1995). Altogether, this evidence indicates that the motor map can flexibly change size in both directions, i.e., increases and decreases, which is indirect biomarker of plasticity attributed to the unmasking of lateral connections of corticospinal neurons (Huntley, 1997). In other words, changes in motor map reflect activity‐dependent modifications of synaptic strength of horizontal connections (Buonomano & Merzenich, 1998; Huntley, 1997).

Enlargement of the motor map area after acquiring motor skills has mainly been demonstrated in the muscles that are primarily involved in the skilled motor task (Kleim et al., 2007; Pascual‐Leone et al., 1993, 1995; Yarossi et al., 2019). It has also been shown that the enlargement of the motor map area in parallel with that of other neighboring muscles (Tyč et al., 2005). This is quite plausible because the functional movements of humans usually comprise synergistic movements of multiple joints that are produced by activities in multiple muscles. Accordingly, many studies have investigated plastic changes in the motor maps of multiple muscles simultaneously after motor training (for review see Sondergaard et al., 2021). However, a recent review of TMS mapping showed that most studies only targeted muscles located within a single segment, such as intrinsic hand muscles, or forearm muscles (Sondergaard et al., 2021). Only a limited number of studies have attempted the simultaneous investigation of changes in the motor map area size of muscle groups beyond segments, such as the shoulder and intrinsic hand muscles (Brasil‐Neto et al., 1992; Devanne et al., 2002, 2006; Wassermann et al., 1992).

One possible reason for this could be that the stimulation intensity for activation of the proximal (e.g., shoulder) and distal (e.g., hand) muscles by TMS may differ substantially, due to their different sensitivities to external stimuli (Siebner & Rothwell, 2003). In most cases, the sensitivity of proximal muscles tends to be lower than that of distal muscles. In this case, activation of the proximal muscles may not be sufficient when the stimulation intensity is not high enough, whereas excessive intensity might cause over‐activation in the distal muscles, leading to a map area size close to the ceiling. In both cases, inappropriate stimulation intensities may fail to detect changes in the map area size which has the potential to change either towards an increase or a decrease. To assess changes in the motor map area size, it is necessary to apply the stimulation intensity at an optimal strength that produces a motor map with sufficient scope for change in both directions (i.e., optimal area size), to be used as a baseline reference.

In the present study, we investigated which groups of muscles could be assessed simultaneously, that is, from which groups of muscles across segments, we could obtain an optimal motor map size (i.e., optimal area size defined as 25%–75% of the maximum area size) and at which level of stimulation intensity it could be achieved. To address these questions, we assessed the motor map of seven muscles simultaneously across multiple segments in the shoulder, upper arm, forearm, and hand, using five different predetermined TMS intensities. We calculated parameters of motor maps (i.e., area, volume, and COG) of each muscle at each stimulation intensity. In this study, we particularly focused on motor map area which is more reliable than motor map volume (Nazarova et al., 2021) and known to be related with motor skill learning and functional improvements as mentioned above. We computed the range of stimulation intensity that produced motor map area with an optimal area size for each muscle and determined the optimal settings at which the optimal area size was obtained for many muscles in as many participants as possible.

## 2 METHODS

### 2.1 Participants

Fifteen healthy young adults (one female; mean age 29.3 years, standard deviation [SD] 5.1 years) participated in this study. All participants were right‐handed according to the Edinburgh Handedness Inventory (Oldfield, 1971) (laterality index: mean 90.4, SD 21.4). None of the participants had contraindications for TMS (Rossi et al., 2009).

All participants provided written informed consent for participation in accordance with the Declaration of Helsinki of 1964, as revised in 2013. This study was approved by the Fujita Health University Certified Review Board (approval no. HM20‐257).

### 2.2 Electromyography

We recorded surface electromyography (EMG) from the following seven muscles of the right upper limb: deltoid anterior (DA), triceps brachii (TB), biceps brachii (BB), flexor carpi radialis (FCR), extensor carpi radialis (ECR), abductor digiti minimi (ADM), and first dorsal interosseous (FDI) muscles. Pairs of adhesive Ag–AgCl surface EMG electrodes (NM‐31; Nihon Kohden Corporation) were placed on the muscle belly of each muscle identified by manual palpations, and the skin was abrased with gel and cleaned with rubbing alcohol. The EMG activities were recorded using a bio‐signal recording system (Nuropack X1 MEB‐2312; Nihon Kohden Corporation) at a frequency of 5 kHz, with band‐pass filtering from 10 Hz to 10 kHz. The recorded data were digitized using a micro 1401 AD converter (Cambridge Electronic Design) and stored on a computer.

### 2.3 Transcranial magnetic stimulation

**FIGURE 1:**
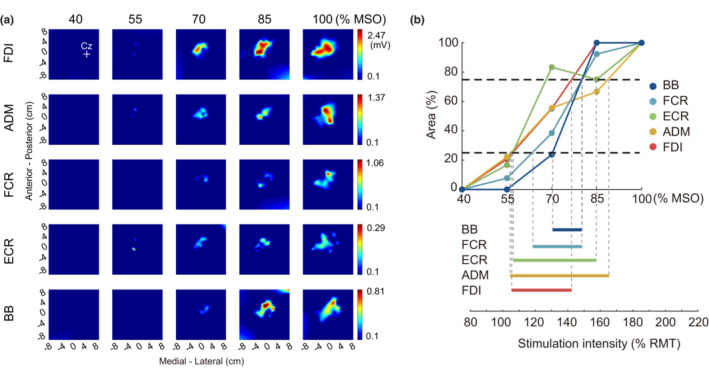
(a) Motor maps of a representative participant acquired at five different stimulation intensities. The coordinate (0, 0) indicates 5 cm to the left of the vertex (Cz). The colors in the maps represent the amplitude of the MEP. (b) The area sizes of motor maps of targeted muscles acquired at five different stimulation intensities in a representative participant. The area size of each muscle (y‐axis) is normalized to the maximum size acquired at a certain stimulation intensity. The range between the two black horizontal dashed lines, representing 25% and 75% of the maximum, indicates an “optimal” area size and the range of stimulation intensity (a range between the two gray vertical dashed lines) that produces the optimal area size is defined as an “optimal” intensity. The intensity % MSO is converted to % RMT of the FDI muscle. Note that motor maps of the deltoid anterior and triceps brachii are not shown because of a lack of MEP samples in this participant. ADM, abductor digiti minimi; BB, biceps brachii; ECR, extensor carpi radialis; FCR, flexor carpi radialis; FDI, first dorsal interosseous; MEP, motor evoked potential; MSO, maximum stimulator output; RMT, resting motor threshold.

Motor mapping was conducted using a 70‐mm‐diameter figure‐of‐eight coil, which was connected to the BiStim^2^ magnetic stimulator (Magstim Company) and positioned at an angle of 45°toward the contralateral forehead to induce the current in the brain in the posterior–anterior direction (Brasil‐Neto et al., 1992; Cavaleri et al., 2018), with a monophasic waveform. Participants were instructed to keep their muscles relaxed during TMS. First, we set the stimulation intensity with an estimated suprathreshold strength to find the optimal stimulation site (“hotspot”) that evoked the largest motor evoked potential (MEP) from the right FDI muscle. We delivered a single pulse to several random locations around 5 cm lateral from the Cz in the left primary motor cortex. We identified the candidate site at which the MEPs were consistently observed with suprathreshold intensity. Then, we delivered two to three stimuli to each of four direction sites (north, east, south, and west) around the candidate site at a distance of approximately 1 cm. Once greater MEP responses were consistently observed than other sites for a given stimulation intensity, we defined the site as the hotspot and marked the location using the custom‐made navigation system written in LabView (National Instruments Corporation) which can record stimulation locations. We then determined the resting motor threshold (RMT) of the FDI muscle, that is, the lowest intensity that evoked a peak‐to‐peak MEP amplitude exceeding 50 μV in at least five of 10 successive stimuli at the hotspot (Rossini et al., 2015). Thereafter, motor mapping was performed using a custom‐made coil‐tracking system with an object‐tracking camera (OptiTrack V120: Duo, Acuity). Using the pseudorandom walk method (Cavaleri et al., 2018; Van De Ruit et al., 2015), we delivered stimuli to random locations with approximately 1 cm apart within a pre‐set area of 16 × 16‐cm square centered 5 cm lateral to the left from the vertex (Cz) (Figure 1a). The coil orientation was visually displayed and maintained at an angle of approximately 45° to the medial sagittal plane throughout the measurement (Janssen et al., 2015). During the mapping procedure, the custom‐made program was used to compute and visualize the estimated motor map area of each muscle based on the spatial information of the stimulated sites and MEP amplitudes. The stimulation sites with MEP amplitudes exceeding 50 μV were plotted with yellow dots, while the others were plotted with green dots, and the border of the yellow and green dots was outlined in red as an estimated motor map area. During the first 60 stimuli, the visual feedback of MEPs of the FDI muscle was provided so that the experimenter could deliver stimuli densely distributed within and around the estimated motor map area and avoid providing extra stimulation in the irrelevant area. Following the previous finding that reliable maps can be obtained with approximately 60 stimuli (Van De Ruit et al., 2015), we temporarily evaluated the shape of the estimated map area of each muscle after delivering 60 stimuli. We only delivered further stimuli if a low‐density area remained. Eventually, we delivered 66.6 stimuli on average (89 stimuli at maximum).

### 2.4 Experimental protocol

The participants were comfortably seated on a chair with a backrest and their forearms placed on cushions for relaxation. To elucidate the optimal TMS intensity that could produce the optimal area size defined as 25%–75% of the maximum motor map area size for each muscle, we performed five sessions of mapping procedures with five different TMS intensities at 40%, 55%, 70%, 85%, and 100% of the maximum stimulator output (MSO). Their order was randomized across participants. During each session, EMG of seven muscles were recorded simultaneously. Each session took 5–8 min; total assessment time was approximately 40 min at maximum (8 min × 5 stimulation intensities).

### 2.5 Data analysis

All analyses were performed offline using a custom program written in MATLAB (MathWorks, Inc.). The peak‐to‐peak MEP amplitudes measured for each muscle were calculated. The EMG recordings were visually inspected and trials with significant cable artifacts and background activity within 100‐ms window prior to the TMS pulse were discarded. As suggested in a previous study (Van De Ruit et al., 2015), outliers that the peak‐to‐peak MEP amplitudes exceeded the mean ± 3.5 SD of all MEP amplitudes in each muscle in each condition (stimulation) were excluded to avoid skewing the map. Consequently, 4.8% of all the collected MEPs among all participants were excluded before constructing the motor map. The motor map was then constructed from the peak‐to‐peak amplitudes of MEPs using a MATLAB surface fitting tool (“gridfit” D'Errico, 2016) to define 2500 partitions (50 × 50) within a 16 × 16‐cm area (Van De Ruit et al., 2015) (Figure 1a). The motor map area was calculated as the ratio of the number of partitions where the approximated MEP amplitudes exceeded 0.1 mV relative to all partitions (Cavaleri et al., 2018; Milosevic et al., 2021; Van De Ruit et al., 2015).

To determine the optimal TMS intensity that could produce a motor map area with an optimal area size, we first calculated the area of the map (cm^2^) acquired at each stimulation intensity relative to the maximum area size to control for variation in the size of the motor map area among participants. Then, we identified the range of TMS intensity that could produce the optimal area size, defined as 25%–75% of the maximum area size, which had sufficient scope to allow an increase or a decrease in motor map area, for each muscle (Figure 1b). The TMS intensity was expressed as the percentage of RMT of the FDI muscle in each participant. Then, we counted the number of participants who showed the optimal area size of the motor map area for each muscle at every 20%‐step in stimulation intensity, from 80% to 240% of RMT. Finally, we determined the optimal range of stimulation intensity by which the optimal area size was obtained for as many participants as possible in a large number of muscles.

In addition, we investigated the motor map volume, another representative parameter of representation mapping, and COG to verify if acquired mapping data were reliable (see supplementary materials for detailed methodology).

## 3 RESULTS

### 3.1 Motor map area

**FIGURE 2:**
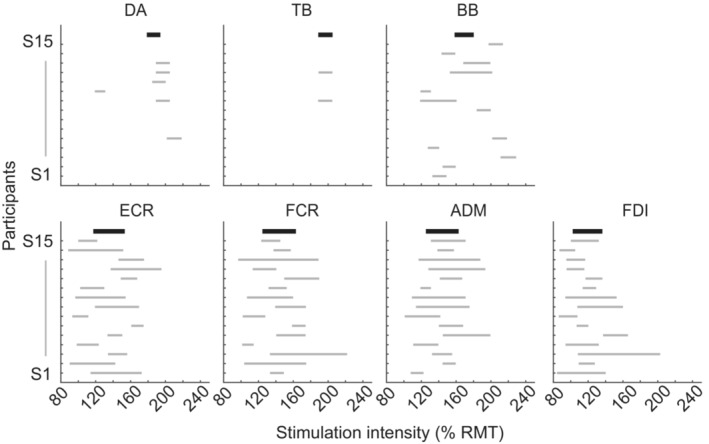
The range of stimulation intensity resulting in the optimal area size (from 25% to 75% of the maximum) of motor maps in each muscle for each participant. The range for each participant is represented by horizontal thin gray lines. The top thick black lines represent the mean across participants. The horizontal and vertical axes represent the stimulation intensity as a percentage of RMT of the FDI muscle and the individual participants, respectively. ADM, abductor digiti minimi; BB, biceps brachii; DA, deltoid anterior; ECR, extensor carpi radialis; FCR, flexor carpi radialis; FDI, first dorsal interosseous; RMT, resting motor threshold; TB, triceps brachii.

Figure 2 shows the range of stimulation intensity that could produce a motor map with an optimal area size (i.e., from 25% to 75% of the maximum area size). The optimal area size of the motor map of the four distal muscles (FCR, ECR, ADM, and FDI) was obtained from all participants with a relatively low‐intensity range. In contrast, the optimal area size of the motor map for the three proximal muscles (DA, TB, and BB) was obtained only in some participants, even when using high stimulation intensities. Notably, the optimal area size of the motor map for the DA and TB muscles was produced in only six and two participants, respectively, and high stimulation intensities were needed. Although the optimal area size of the motor map for the BB was obtained in a relatively greater number of participants than that for the rest of the proximal muscles, the stimulation intensity required varied widely among participants.

**FIGURE 3:**
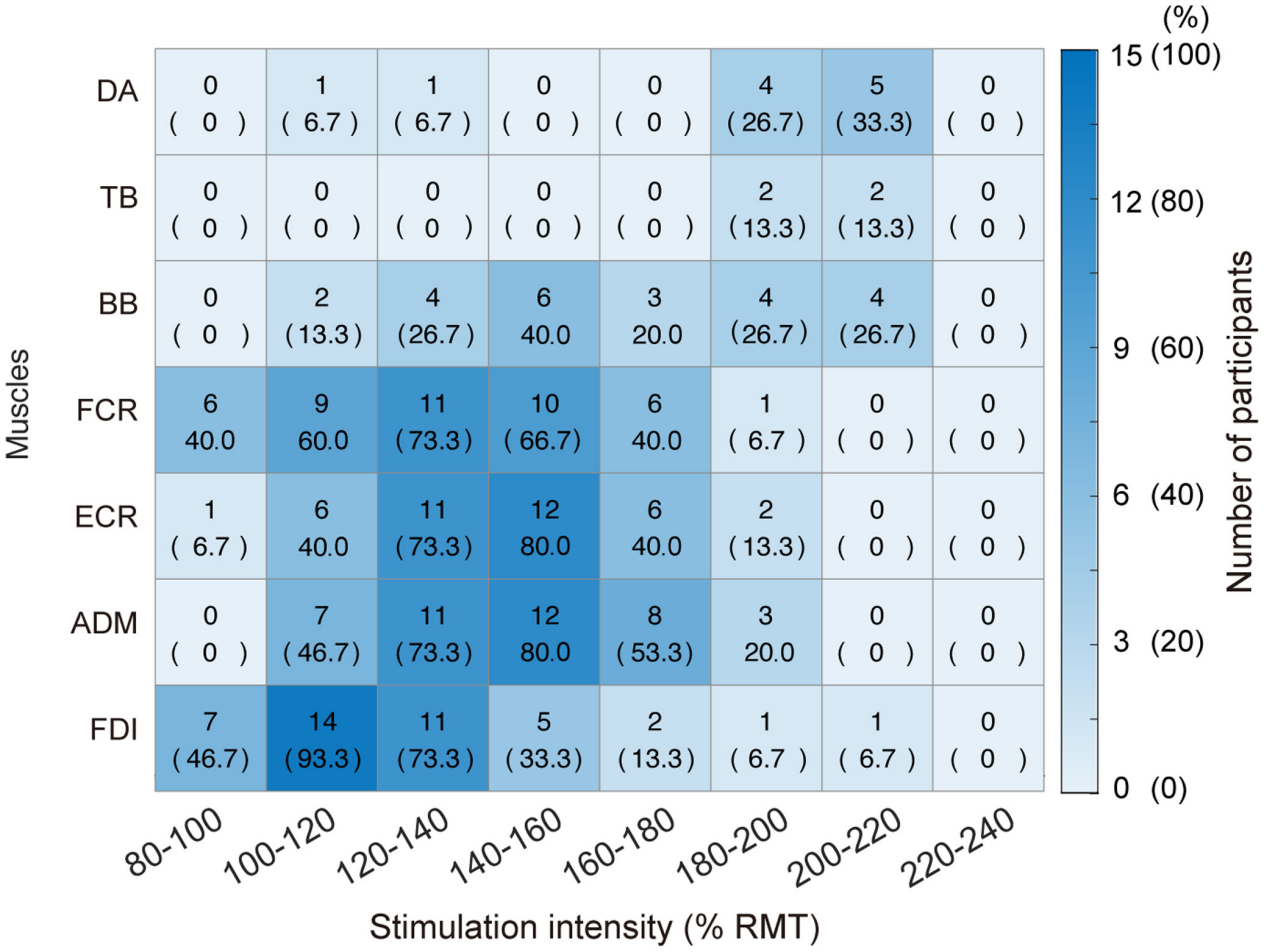
The number of participants who showed the optimal motor map area size at every 20%‐step of stimulation intensity (% RMT). Data for each muscle are aligned in rows. The number in brackets indicates the percentage of participants. The number/percentage of the participants is also represented in colors, with the greater number in darker blue. ADM, abductor digiti minimi; BB, biceps brachii; DA, deltoid anterior; ECR, extensor carpi radialis; FCR, flexor carpi radialis; FDI, first dorsal interosseous; RMT, resting motor threshold; TB, triceps brachii.

Regarding the number of participants who showed the optimal area size at every 20%‐step in TMS intensity (Figure 3), we found that the optimal area size of the motor maps for the four distal muscles (FCR, ECR, ADM, and FDI) was produced in most participants (5–14 participants: 33.3%–93.3% of all participants) when the stimulation intensity was set at a range of 100%–160% of RMT. Specifically, when the stimulation intensity was set in the range of 120%–140% of RMT, the optimal area size of the motor maps of those muscles was produced in the largest number of participants (11 participants: 73.3% of all participants). For the proximal muscles (DA, TB, and BB), the optimal area size of the motor map for all three these muscles was produced only in a few participants (2–5 participants; 13.3%–33.3% of all participants), and required a higher stimulation intensity, from 180%–220% of RMT. Importantly, no stimulation intensity simultaneously produced the optimal area size of motor maps for all seven muscles across segments. Although a stimulation intensity of 180%–200% of RMT was able to produce the optimal area size of the motor map from at least one participant in all seven muscles, the number of participants was small (1–4 participants), and no single participant showed an optimal area size of the motor maps for all muscles simultaneously with that range of intensity (Figures S1 and S2).

### 3.2 Motor map volume

We found that the optimal volume size of the motor maps for the four distal muscles (FCR, ECR, ADM, and FDI) was produced in most participants (8–14 participants: 53.3%–93.3% of all participants) when the stimulation intensity was set at a range of 140%–160% of RMT (Figure S3). As found in the motor map area, the optimal volume size for the proximal muscles (DA, TB, and BB) was produced only in a few participants (2–5 participants; 13.3%–33.3% of all participants), and required a higher stimulation intensity, from 180%–220% of RMT.

### 3.3 Center of gravity

There were no significant differences between intensities in any muscles in both of x‐ and y‐ coordinates (mediolateral and anteroposterior axes, respectively) except for y‐coordinate of the FCR muscle in both of x‐ and y‐ coordinate (Figure S4 and Table S1). This additional result indicates that the mapping data was reliably obtained across sessions.

## 4 DISCUSSION

TMS mapping in a short time is essential to capture a short‐lasting change in the neural excitability in the motor cortex after certain events. Thus, a reliable procedure for simultaneous mapping of multiple muscles should be explored. To achieve it, the present study set the optimal area size (defined as 25%–75% of the maximum area) and systematically investigated in which muscle groups across segments it was possible to obtain motor maps with optimal area sizes simultaneously, and which level of stimulation intensity was required to achieve this. We found that the optimal area size could not be obtained for motor maps from all targeted muscles simultaneously when using the same level of stimulation intensity. However, when limiting our investigations to the four distal muscles in the forearm and hand, the optimal area size for these motor maps was produced at the same level of stimulation intensity: 120%–140% RMT of the FDI muscle. Although motor maps with an optimal area size could be obtained for a group of proximal muscles in the shoulder and upper arm simultaneously, a markedly higher stimulation intensity (180%–220% of RMT) was required, and the optimal area size was produced in only a few participants.

A limited number of previous studies have investigated the cortical representations of multiple muscles from the shoulder to the hand and have demonstrated that motor maps from all the targeted muscles across segments were produced only when using the 100% of MSO (Brasil‐Neto et al., 1992; Wassermann et al., 1992). This indicates that motor maps can be obtained for muscles across segments by using a high stimulation intensity. However, using this may cause a ceiling effect in the motor map area of the distal muscles, the RMT of which is usually low and thus sensitive to external electrical stimulation. Because the motor map flexibly changes in area size (increasing or decreasing, depending on the situation), saturation of the motor map area might lead to a failure to detect plastic changes. To address this issue, we defined the optimal area size of the motor map with sufficient scope in both directions (i.e., 25%–75% of the maximum) as a criterion for assessing motor maps. Consequently, contrary to previous studies (Brasil‐Neto et al., 1992; Wassermann et al., 1992), we found that motor maps with optimal area sizes could not be obtained for all targeted muscles simultaneously, even when using 100% of MSO. Instead, our results demonstrated that the optimal motor maps can be obtained simultaneously for the group of distal muscles in the forearm and hand, while they can be obtained separately for the proximal muscles in the shoulder and upper arm, likely due to the difference in required stimulation intensities for the proximal and distal muscles.

Anatomical aspects identified in previous physiological experiments offer several possible explanations for the difference in the stimulation intensity required to produce motor maps with an optimal area size for the various muscle groups. First, the strength of corticospinal projections differs among muscles. In non‐human primates, it has been revealed that the number of corticomotoneuronal cells that directly terminate in motoneuron pools is greater in distal muscles than in proximal muscles (McKiernan et al., 1998). In humans, a large proportion of corticomotoneurons project to the distal hand muscles, followed by the forearm and BB muscles, whereas there are few corticomotoneuronal projections to proximal muscles (Brouwer & Ashby, 1990; Palmer & Ashby, 1992). Related to these findings, it has been demonstrated that proximal muscles are mainly controlled by ventromedial brainstem pathways that project bilaterally and enable coordinated movements with trunk muscles, while distal muscles are controlled by dorsolateral brainstem pathways, mainly unilateral projections of the corticospinal tracts (for review see Lemon, 2008). Since TMS activates pyramidal tract neurons indirectly (transsynaptically) and as muscle responses (MEPs) reflect the sum of neural activity of corticospinal neurons (Siebner & Rothwell, 2003; Terao & Ugawa, 2002), the strong corticospinal projections to the motoneurons innervating the targeted muscle would lead to a large response to TMS. Second, the sensitivity of cortical microstructures responsible for MEPs may differ between regions projecting to the proximal and to the distal muscles. MEPs are comprised of multiple indirect waves, named I‐I, I‐II, and I‐III, which are attributed to transsynaptic activation of pyramidal tract neurons (Terao & Ugawa, 2002). Since I‐I waves tend to be provoked by relatively weak external stimulation (Nakamura et al., 1996; Terao & Ugawa, 2002), MEPs elicited by low‐intensity TMS mainly reflect the summation of I‐I waves. A previous study suggested that the proportion of I‐I waves in the biceps is lower than that in the FDI (Martin et al., 2006). This indicates that proximal muscles are less sensitive to TMS than distal muscles. These physiological differences between muscles may underlie the differences in stimulation intensities required to produce the optimal area size of the motor maps. Moreover, this may contribute to the finding that only a few participants exhibited motor maps with optimal area size for the proximal muscles, even at a high stimulation intensity. It remains unclear whether motor maps can be produced when the stimulator output is increased beyond the maximum of the machine used in this study.

There is also another issue if the stimulation intensity is not high enough, the activation of the neurons is insufficient, which might also cause the failure to capture the changes in motor maps. To address this, we set the optimal area size with sufficient scope in decrease as greater than 25% of maximum area size. To date, the stimulation intensity of 110% or 120% RMT of the intrinsic hand muscles such as the FDI and ADM muscles has been often used in motor mapping for not only a single muscle but also for multiple muscles (Sondergaard et al., 2021). Contrary to this conventional method, our results suggest that relatively higher stimulation intensity (i.e., 120%–140% RMT of FDI) is appropriate to capture the changes of motor map area for simultaneous mapping of multiple muscles especially for distal forearm and hand muscles. In accordance with the present finding, previous studies suggested that a higher stimulation intensity is needed for performing motor mapping of multiple muscles when using the intensity based on RMT of a hand muscle as a reference intensity. For example, Yarossi et al. (2019) investigating training effects in people after stroke with motor maps using110% RMT of FDI and showed significant enlargement of the motor map area in the trained finger muscles but not in the forearm muscles. They interpreted the results that the stimulation intensity was too weak to capture the plastic changes of the forearm muscles. Furthermore, a recent study by Jin et al. (2022) tested the reliability of motor map area of eight finger and forearm muscles simultaneously using different stimulation intensities. They showed good reliability in three muscles using 105% RMT of FCR, while moderate to poor reliability in all eight muscles using lower stimulation intensity of 105% RMT of FDI. Thus, it seems that the slightly higher stimulation intensity might result in more reliable motor map. Together with the previous evidence, the present study demonstrates that relatively higher stimulation intensity would be appropriate for successfully performing simultaneous mapping of multiple muscles.

Regarding the motor map volume, our results showed that the optimal volume size was produced for three proximal muscles (DA, TB, and BB) and for four distal muscles (FCR, ECR, ADM, and FDI) separately using different stimulation intensity as found in the motor map area. However, the optimal TMS intensity for the distal muscles to produce the optimal volume size was relatively higher (i.e., 140%–160% RMT of FDI) than that to produce the optimal area size. This difference can be explained by the assumption that the motor map volume does not reflect the topography of the representation of a particular muscle but the overall excitability of the cortical representation (Rossini et al., 2015). Given that the motor map volume includes variables of the number of locations where MEPs are elicited and the MEPs amplitude at each location, higher stimulation intensity is needed to make the motor map volume reach the maximum than that for the area size. Therefore, the stimulation intensity required to obtain the optimal size (25%–75% of the maximum) of the motor map volume might shift to a higher range compared to that of the motor map area.

This study has a limitation that the study was conducted only with healthy young participants. Therefore, it is uncertain whether our findings are directly transferable to older individuals and to those with neurological disorders. It has been reported that older people show higher motor thresholds and smaller MEP amplitudes than younger participants (Bashir et al., 2014), partly due to the age‐related decrease in the number of corticomotor neurons or their dysfunction (Eisen et al., 1996). In stroke patients, the motor threshold in the ipsilesional motor cortex increases because of damage to the corticospinal tract and/or other pathophysiological conditions, such as edema and metabolic dysfunction (Xing et al., 2012). This suggests that higher stimulation intensity may be required for the distal and proximal muscles. Moreover, it may be more difficult to obtain motor maps of the proximal muscles in these populations than in healthy young people. Further research is required to address this possibility.

## 5 CONCLUSION

The results of this study suggest that motor maps can be assessed in the forearm and intrinsic hand muscles simultaneously, using the same level of TMS intensity, whereas a higher stimulation intensity is required for obtaining suitable motor maps of the shoulder and upper arm muscles. Therefore, separate motor mapping may be necessary for the proximal and distal muscles, with different experimental settings. These findings provide insight into methods for precisely assessing plastic changes in motor representation after certain events, such as after lesioning of the nervous system and after acquisition of skilled movements.

## FUNDING INFORMATION

This work was supported the Japan Society for the Promotion of Science (JSPS) Grants‐in‐Aid for Scientific Research (KAKENHI) [20 K23298].

## CONFLICT OF INTEREST

The authors have no conflict of interest.

## ETHICS APPROVAL

Approval was obtained from the Certified Review Board at Fujita Health University (approval No. HM20‐257). All participants provided written informed consent before participation according to the Declaration of Helsinki of 1964, as revised in 2013.

## Supporting information


Figure S1.

Figure S2.

Figure S3.

Figure S4.

Appendix S1:
