# Supplementary figures and images for: Systematic determination of muscle groups and optimal stimulation intensity for simultaneous TMS mapping of multiple muscles in the upper limb
Source: Physiol Rep. 2022 Dec 2;10(23):e15527. doi: 10.14814/phy2.15527 (PMC9718942; doi:10.14814/phy2.15527)

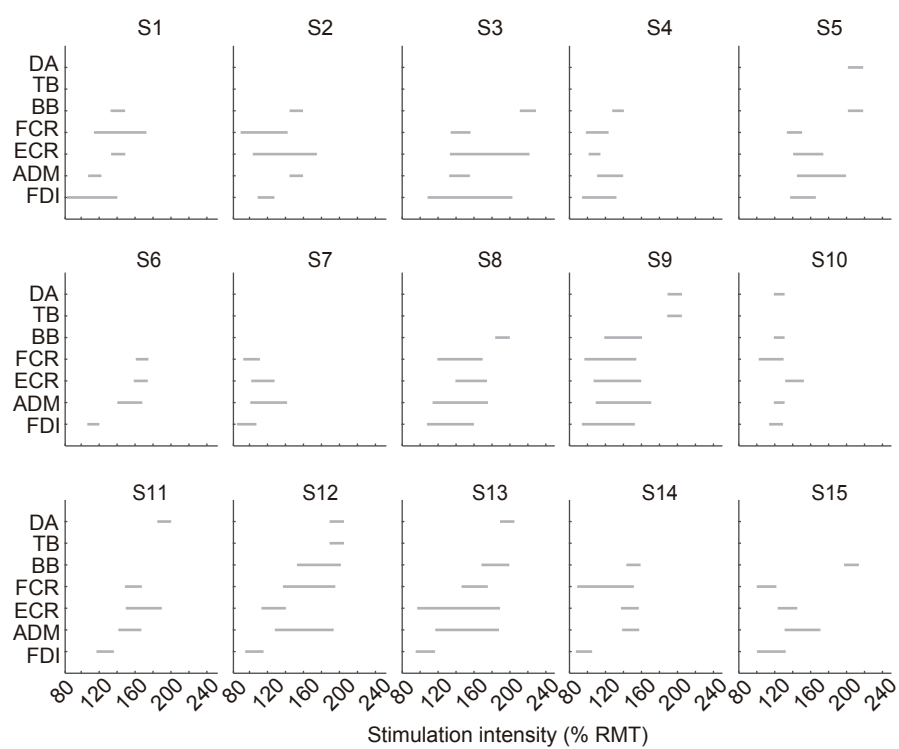

Supplement: Supplementary file 1 — Figure S1. [file PHY2-10-e15527-s001.pdf]

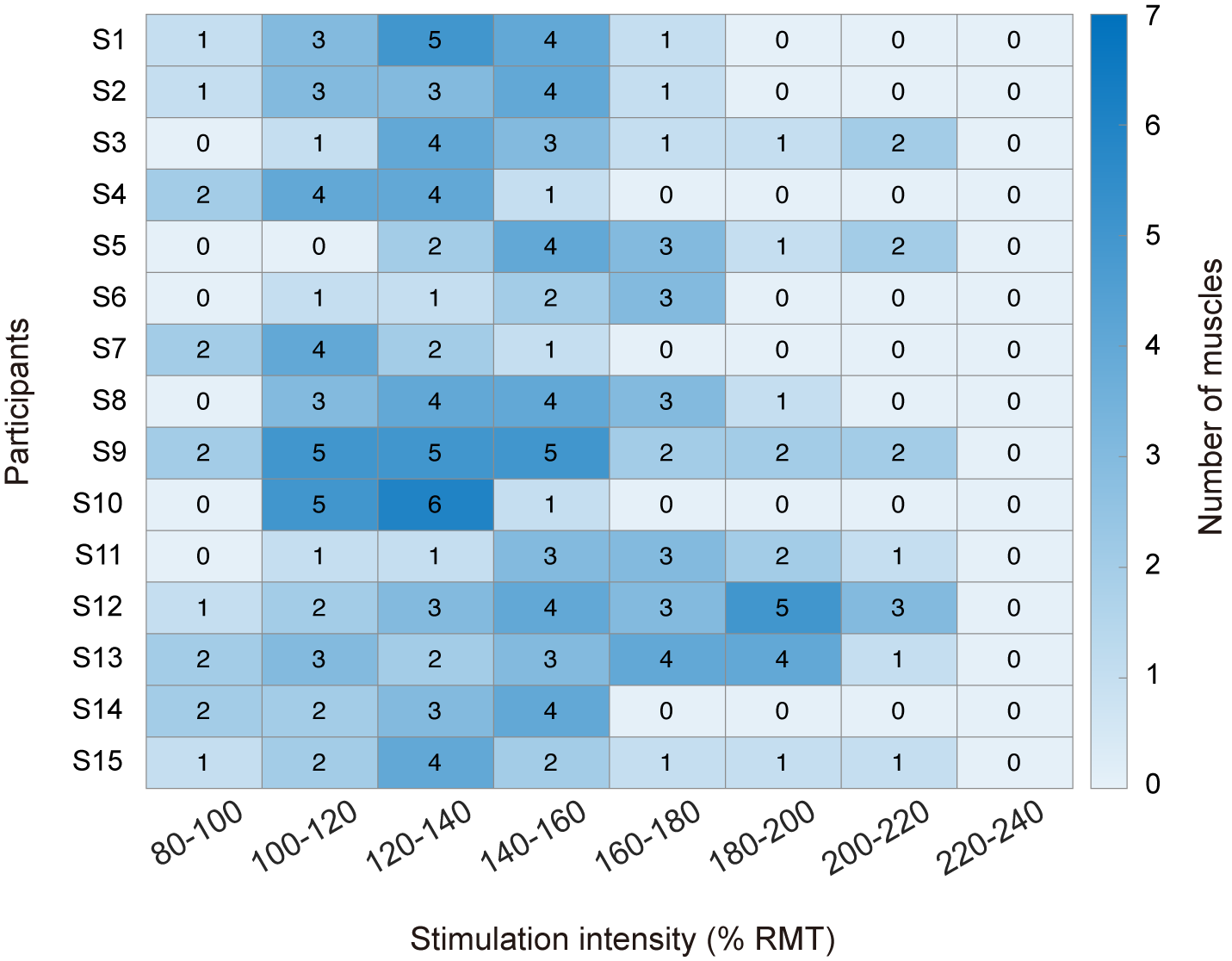

Supplement: Supplementary file 2 — Figure S2. [file PHY2-10-e15527-s005.tif]

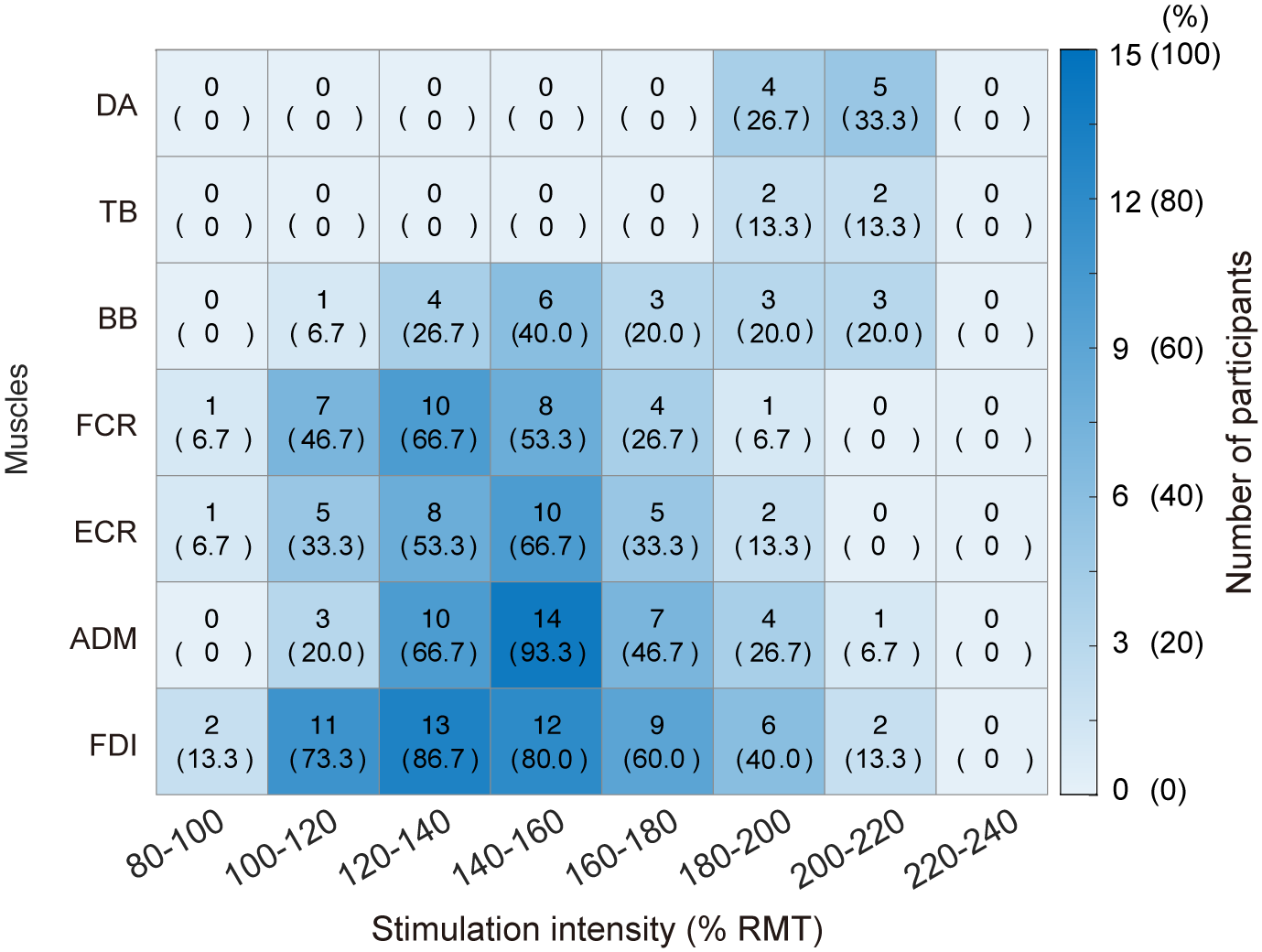

Supplement: Supplementary file 3 — Figure S3. [file PHY2-10-e15527-s003.tif]

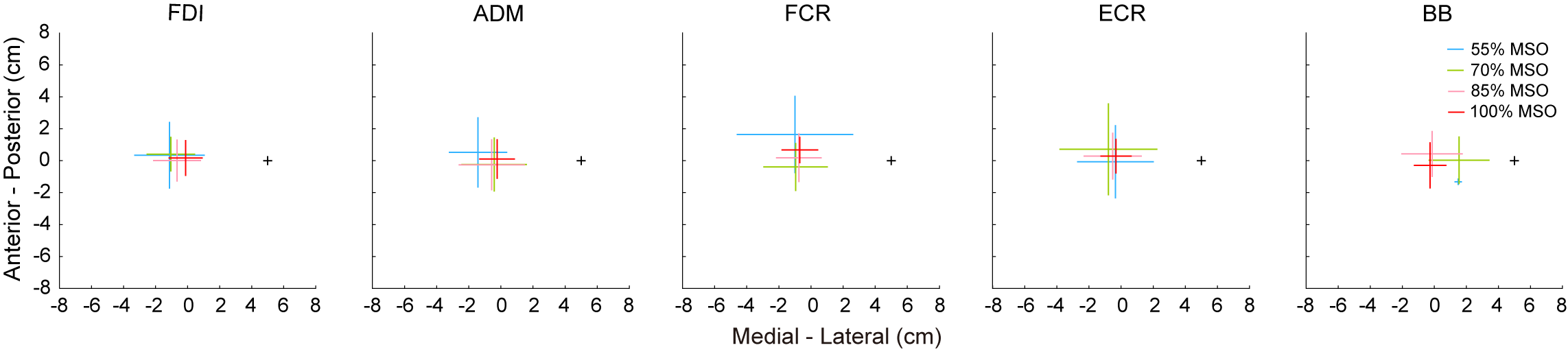

Supplement: Supplementary file 4 — Figure S4. [file PHY2-10-e15527-s002.tif]
