# Supplementary material for: Systematic determination of muscle groups and optimal stimulation intensity for simultaneous TMS mapping of multiple muscles in the upper limb
Source: Physiol Rep. 2022 Dec 2;10(23):e15527. doi: 10.14814/phy2.15527 (PMC9718942; doi:10.14814/phy2.15527)
Supplement: Supplementary file 5 — Appendix S1: [file PHY2-10-e15527-s004.docx]

**Supplementary material:**

**Motor map volume**

We calculated the motor map volume as the sum of all approximated MEPs amplitude exceeding 0.1 mV, subtracted by the 0.1 mV multiplied by the number of these MEPs, and then normalized to the largest MEP amplitude of each muscle (Rossini et al., 2015; Van De Ruit, Perenboom and Grey, 2015). Then, as for the motor map area, we identified the optimal TMS intensity that could produce the optimal volume size, defined as 25%－75% of the maximum volume size.

**Center of gravity**

We calculated COG as a weighted average of the location (Van De Ruit, Perenboom and Grey, 2015), then compared the coordinates of COG (x- and y- coordinate separately) between intensities in each muscle using mixed effects model with stimulation intensity as a fixed effect and participant as a random effect. We excluded the session with 40% MSO in which we could not obtain MEP responses in most of participants. Note that we focused only on COG data of the five muscles other than the proximal triceps and deltoid muscles that had only a few data samples.

Table S1. Summary table of mixed effects model of the center of gravity.

| *P*-values | FDI | ADM | FCR | ECR | BB |
| --- | --- | --- | --- | --- | --- |
| x-coordinate | 0.109 | 0.451 | 0.976 | 0.933 | 0.066 |
| y-coordinate | 0.848 | 0.679 | 0.020 | 0.809 | 0.403 |

Abbreviations: ADM, abductor digiti minimi; BB, biceps brachii; ECR, extensor carpi radialis; FCR, flexor carpi radialis; FDI, first dorsal interosseous.

Table S2. Terms and their definitions used in the present study.

| Terms | Definition |
| --- | --- |
| Motor map area | The part of the map where the approximated MEPs amplitude exceeds a predefined threshold (i.e., 0.1 mV). |
| Maximum area size | The maximum size of the motor map area of each muscle obtained when stimulated by either stimulation intensities of 40, 55, 70, 85, and 100 % of MSO). |
| Optimal area size | 25%–75% of the maximum area size of each muscle, which has sufficient scope to allow an increase or a decrease in motor map area. |
| Motor map volume | The sum of all approximated MEPs amplitude exceeding 0.1 mV, subtracted by the 0.1 mV multiplied by the number of these MEPs, and then normalized to the largest MEP amplitude. |
| Maximum volume size | The maximum size of the motor map volume of each muscle when stimulated by either stimulation intensities of 40, 55, 70, 85, and 100 % of MSO). |
| Optimal volume size | 25%–75% of the maximum volume size of each muscle, which has sufficient scope to allow an increase or a decrease in motor map area. |
| Optimal TMS intensity | The 20% range of stimulation intensity that can produce the optimal area/volume size as many participants as possible in a large number of muscles simultaneously. The intensity is expressed as the percentage of RMT in the FDI muscle in each participant. |

Abbreviations: FDI, first dorsal interosseous; MEP, motor evoked potential; MSO, maximum stimulator output; RMT, resting motor threshold; TMS, transcranial magnetic stimulation.
